# HIV-1 Infection of T Lymphocytes and Macrophages Affects Their Migration *via* Nef

**DOI:** 10.3389/fimmu.2015.00514

**Published:** 2015-10-06

**Authors:** Christel Vérollet, Véronique Le Cabec, Isabelle Maridonneau-Parini

**Affiliations:** ^1^CNRS UMR 5089, Institut de Pharmacologie et de Biologie Structurale (IPBS), Toulouse, France; ^2^Institut de Pharmacologie et de Biologie Structurale (IPBS), Université Toulouse III – Paul Sabatier, Toulouse, France

**Keywords:** macrophages, HIV-1, Nef, cell migration, podosomes

## Abstract

The human immunodeficiency virus (HIV-1) disseminates in the body and is found in several organs and tissues. Although HIV-1 mainly targets both CD4^+^ T lymphocytes and macrophages, it has contrasting effects between these cell populations. HIV-1 infection namely reduces the viability of CD4^+^ T cells, whereas infected macrophages are long-lived. In addition, the migration of T cells is reduced by the infection, whereas HIV-1 differentially modulates the migration modes of macrophages. In 2-dimensions (2D) assays, infected macrophages are less motile compared to the control counterparts. In 3D environments, macrophages use two migration modes that are dependent on the matrix architecture: amoeboid and mesenchymal migration. HIV-1-infected macrophages exhibit a reduced amoeboid migration but an enhanced mesenchymal migration, via the viral protein Nef. Indeed, the mesenchymal migration involves podosomes, and Nef stabilizes these cell structures through the activation of the tyrosine kinase Hck, which in turn phosphorylates the Wiskott–Aldrich syndrome protein (WASP). WASP is a key player in actin remodeling and cell migration. The reprogramed motility of infected macrophages observed *in vitro* correlates *in vivo* with enhanced macrophage infiltration in experimental tumors in Nef-transgenic mice compared to control mice. In conclusion, HIV infection of host target cells modifies their migration capacity; we infer that HIV-1 enhances virus spreading in confined environments by reducing T cells migration, and facilitates virus dissemination into different organs and tissues of the human body by enhancing macrophage mesenchymal migration.

## Introduction

The biology and pathogenesis of HIV infection has been largely studied since the discovery of the virus in 1983. Although the molecular mechanisms involved in virus internalization and replication within its host cells are well described, other key mechanisms of HIV pathogenesis and progression to AIDS, including virus dissemination, are less characterized. During unprotected sexual intercourse, HIV-1 is mainly transmitted through genital and/or rectal mucosal sites, resulting in rapid infection of target cells and access to the bloodstream to ultimately reach lymph nodes at different sites, including the gut, spleen, and lungs, as well as the brain (1). Different mechanisms are involved in HIV-1 dissemination, such as a short period of time when cell-free virus circulates in body fluids and a phase in which the virus can be transported by carrier cells. HIV-1 targets CD4^+^ T lymphocytes and cells from the mononuclear phagocyte lineage (i.e., monocytes, macrophages, and dendritic cells). The virus developed several strategies to disseminate within the human body using host cells. One of the well-documented strategies is cell-to-cell transfer. For instance, T-cell-to-T-cell and macrophage-to-T-cell transfer of the virus is an efficient way for the virus to spread locally (2–6). Another strategy consists in manipulating host cell migration, as shown previously for CD4^+^ T lymphocytes (7), and more recently, for macrophages (8). Regarding HIV-infected T cells, they are able to migrate within lymph nodes favoring virus spreading by a cell-to-cell transfer mechanism (7). Blocking the egress of T cells, or any other cell from the lymph nodes into efferent lymph vessels, decreases viremia. However, it is not established whether this process could account for virus spreading in body tissues (1, 7). Of note, T cells rapidly die after infection in patients. By contrast, macrophages are able to survive to infection and they have the ability to migrate into all body tissues. They are thus suspected to be a target of great interest for HIV to spread. We have recently shown that macrophage migration is reprogramed on infection (8), a mechanism that could contribute to HIV-1 dissemination in the body. Despite the fact that HIV-1 or HIV-1 proteins are well known to induce a “bystander” effect on macrophage migration through the concerted modulation of cytokines/chemokines (9–13); in this review, we focus on the intracellular molecular impact of HIV-1 to control the migration of host cells.

## HIV-1 Nef Regulates Both T Cell and Macrophage Migration

The migration of CD4^+^ T lymphocytes is inhibited by HIV-1 infection both *in vitro* and in lymph nodes (7, 14–18). The HIV-1 protein Nef is responsible for the inhibition of T cell migration *in vitro* both in 2D and in 3D environments (14–18). In addition, Vpu is also an important factor for impaired migration of infected T lymphocytes toward CCL19 (19). HIV-1 infection also markedly affects the migration of human macrophages, as their 2D migration is inhibited and their 3D migration is affected (8). Of note, the 3D migration of macrophages is a complex phenomenon that involves two distinct modes dictated by the extracellular matrix (ECM) architecture (20). In environments with high porosity, macrophages use the amoeboid mode that is characterized by a round cell morphology, the involvement of the ROCK signaling pathway, and a high velocity of cells that squeeze themselves into matrix pores (20). In environments with low porosity, macrophages use the mesenchymal mode that is distinguished by an elongated and protrusive cell morphology, a directed motion with moderate velocity, and the requirement of proteases (20). In the latter case, matrix proteolysis is associated with its ingestion and compaction that allow macrophages to create paths (8, 20–28). In marked contrast with macrophages, all other leukocytes only use the amoeboid mode (29). Interestingly, amoeboid migration is inhibited in HIV-1 infected macrophages while the mesenchymal mode is enhanced (8). These effects of HIV-1 on 2D and 3D macrophage migration are all mediated by Nef. Indeed, the infection of macrophages with a Nef-deleted virus (Δ*nef* HIV-1) lacks all these migration defects, and the targeted expression of Nef in macrophages recapitulates them (8). Therefore, Nef is a master regulator by which HIV-1 modulates all form of motility used by host cells.

Among pathological contexts, malignant tumors represent a tissue model to study the macrophage 3D migration. Indeed, tumor formation triggers the immediate recruitment from tumor-associated macrophages (TAM) that originate of blood monocytes (30). We used this model to assess the impact of HIV-1 on macrophage migration and tissue infiltration to complement our *in vitro* observations using different 3D environments. In Nef-transgenic (Tg) mice, a higher number of TAM is observed compared to those from littermate non-Tg mice. This result may seem surprising since in macrophages Nef inhibits amoeboid and 2D migration and only enhances mesenchymal migration, but it can be reconciliated if the architectural properties of malignant tumors are taken into consideration. Indeed, malignant tumors are dense and rigid (31–33), which likely favor the use of the mesenchymal mode of migration by macrophages. Moreover, we have recently observed that mesenchymal migration is involved in TAM infiltration [Ref. (21) and Gui et al. manuscript in preparation]. Thus, Nef seems to favor *in vivo* macrophage infiltration in dense tissues, such as malignant tumors, possibly by enhancing the mesenchymal mode of migration. In the light of these data, we propose that in porous tissues in which macrophages use the amoeboid mode, Nef-expressing macrophages would be less numerous than control macrophages. In other words, HIV-infected macrophages would accumulate in dense tissues and poorly infiltrate porous tissue regions, allowing virus dissemination preferentially in certain body tissues or in certain area of tissues with heterogeneous architectures. In infected patients and macaques, macrophages are found in several tissues including kidney, liver, gastrointestinal tract mucosa, and brain (34–37). Interestingly, in Nef Tg mice, we observed a high number of macrophages in kidney, liver, and gut tissues as compared to control mice. To better understand these results, it would be informative to characterize the biophysical properties of tissues massively infiltrated by infected macrophages and compare them to those that lack this type of cell accumulation in order to establish a correlation between tissue characteristics and the infiltration of infected macrophages. It would be also interesting to explore whether macrophages are more abundant in the brain from Nef Tg mice, as this critical organ is highly infiltrated by macrophages in both HIV^+^ patients and SIV-infected primates (36). This is an important question since the presence of infected macrophages is highly correlated to neurological dysfunction leading to HIV-1-associated dementia (36, 38–40).

Finally, Nef is also known to inhibit the migration of T cells *in vitro* and block their extravasation through endothelial venules *in vivo* (1, 17). Within lymph nodes, the migration of infected lymphocytes is impaired favoring cell-to-cell contact and possibly resulting in a higher incidence of virus transfer among resident cells (7). In fact, the high cellular promiscuity likely allows HIV-1 to be transmitted both via the *cis*-type and trans-type infection using the formation of virological synapses, as mainly reported *in vitro* (1). Since macrophages exhibit a long-lasting survival, and display a capacity to efficiently migrate and infiltrate all body tissues, we will now focus on the migration mechanisms of the HIV-1-infected macrophages.

## HIV-1 Nef is an Actin Modulator That Targets Podosomes

HIV-1 Nef triggers F-actin remodeling in all cell targets (e.g., T lymphocytes, macrophages, and dendritic cells) (8, 41, 42). Depending on the cell type, Nef can cause depolymerization or polymerization of F-actin (42). In macrophages, Nef induces the formation of F-actin nanotubes to transfer viral particles to B cells (6). In T cells, Nef effects on the cytoskeleton dynamics, and thus help the virus to enter into cells and to transfer into another lymphocyte by the formation of virological synapses, prevent the formation of actin ruffles, and trigger filopodium-like protrusions (15, 18, 42). Cell migration is an actin-dependent mechanism. HIV-1/Nef has pleiotropic effects on HIV-1-infected and Nef-transfected T cell, including its impact on the actin cytoskeleton (42–44). Indeed, Nef is known to inhibit T cell migration by altering the phosphorylation/dephosphorylation of cofilin, which triggers actin filament disassembly (17, 18). Nevertheless, there are no differences in the status of cofilin phosphorylation in *wt*HIV-1, Δ*nef* HIV-1 or uninfected macrophages. Consequently, we infer that the cofilin pathway is not involved in the inhibition of macrophage amoeboid movement.

**Figure 1:**
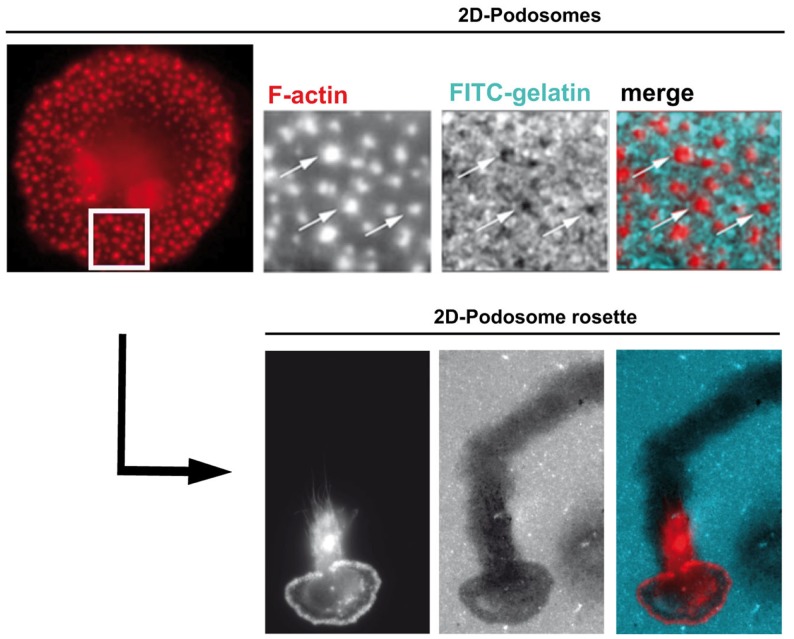
**Human macrophage podosomes in 2D environments**. On 2D surfaces, podosomes stained for F-actin (red) are scattered (upper panels) or organized as rosettes (lower panels). Both individual podosomes and podosomes organized as rosettes are involved in matrix degradation, as shown by dark holes (upper panels, arrows) and a track (lower panels) in the FITC-gelatin (blue) coating. Activation of some podosome proteins favors the organization of podosomes as rosettes and, consequently, increases proteolysis of the extracellular matrix (45, 46).

**Figure 2:**
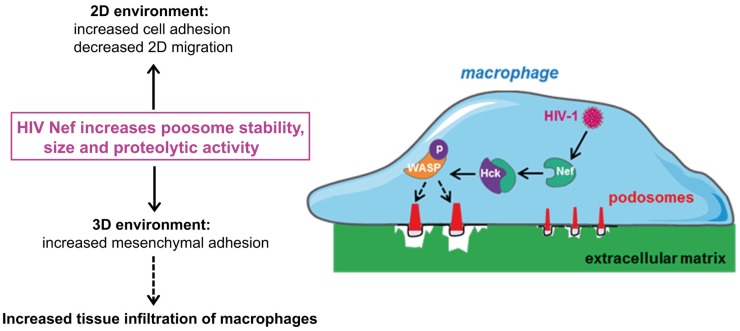
**By targeting podosomes, HIV-1 Nef inhibits 2D migration and enhances the 3D mesenchymal migration mode of macrophages**. HIV-1 infection of macrophages increases the size and stability of podosomes, compared to non-infected cells. The molecular mechanisms responsible for this process involve the interaction of the viral protein Nef with Hck, and WASP phosphorylation. Increase in podosome size correlates with (1) increase in cell adhesion and inhibition of 2D migration and (2) increase in matrix degradation, enhancement of 3D mesenchymal migration and thus macrophage infiltration in tissues.

Podosomes are constitutively formed in few cell types, including macrophages, immature dendritic cells, and osteoclasts when adhering on stiff substrates. These structures are involved in cell adhesion, proteolytic degradation of the ECM, mechanosensing, and mesenchymal migration. They are not formed in cells that use the amoeboid migration, such as T lymphocytes (8, 20–27, 29). When macrophages are plated on coverslips, podosomes assemble at the ventral plasma membrane and are oriented perpendicularly to the substrate. They are constituted of an F-actin core surrounded by a ring of adhesion proteins, and concentrate most of the cellular F-actin. The organization of podosomes into super-structures called “podosome rosettes” is related to an increase in ECM degradation (45, 46) (Figure 1). In HIV-1-infected cells, podosomes become bigger, they assemble into podosome rosettes and the ECM degradation is enhanced, phenomena which are not observed in macrophages infected with Δ*nef* HIV-1. In Nef-expressing macrophages, podosomes are more stable, their life span is doubled, and they degrade very actively the ECM, possibly as a result of their increased lifetime and their rosette organization. Interestingly, Nef accumulates in the podosome area, suggesting that it could interact with a podosome effector regulating the stability of these cell structures. In 3D environments, podosomes assemble at the tip of cell protrusions; they are called 3D podosomes (28). Interestingly, HIV-1-infected cells and Nef-expressing macrophages form more 3D podosomes than control cells (8). The regulation of podosomes by Nef can explain two aspects of the modified migration of macrophages by HIV-1 (Figure 2). First, podosomes that are adhesion cell structures are more stable and bigger, thus explaining the increased cell adhesion observed in HIV-1-infected macrophages (8). Actually, modifications in cell adhesion are known to result in altered 2D cell motility (47, 48). For example, the maturation of dendritic cells, which induces the dissolution of podosomes, allows these cells to undergo the transition from an adhesive to a highly migratory phenotype (49, 50). By contrast, increased cell adhesion by more stable podosomes should decrease 2D migration of HIV-1-infected macrophages. Second, 3D mesenchymal migration correlates with podosome stability and an increase in ECM proteolytic activity (45, 46), two parameters increased by HIV-1 infection (8). Therefore, by affecting podosomes, HIV-1 reduces the macrophage 2D migration and enhances 3D mesenchymal migration. This is the first pathogen known to target podosomes to control the migration of host cells.

## Nef/Hck Interaction: A Potential Pharmacological Target

Nef modulates the function of several proteins also described to localize at podosomes, including Wiskott–Aldrich syndrome protein (WASP), Hck, paxillin, and cofilin (18, 51–53). Hck and WASP are key effectors of the mesenchymal migration in macrophages (25, 45, 54). Hck is a tyrosine kinase of the Src family specifically expressed in phagocytes. Hck controls several processes, such as receptor signaling, lysosome trafficking, podosome stability, and organization into podosome rosettes, as well as proteolytic activity of podosomes (45, 55–57). Interestingly, mesenchymal migration is reduced in Hck^−/−^ or WASP^−/−^ macrophages, while Hck and WASP depletion have no effect on amoeboid migration.

Among a plethora of host proteins, Nef interacts through its poly-proline sequence with the SH3 domain of Src tyrosine kinases (58). In macrophages, this interaction mediates activation of Hck (53). In addition to the poly-proline sequence of Nef, several other Nef domains are involved in podosome regulation suggesting that other Nef effectors than Hck control podosomes. However, Hck appeared to be the main actor for enhancing mesenchymal migration since this effect is abolished when Hck is knocked-down in HIV-1-infected macrophages. These results suggest that Hck plays distinct roles in mesenchymal migration controlling either podosome formation or protease release. Actually, the p61Hck isoform is located at lysosomes and is involved in the proteolytic activity of podosomes (55), which is necessary for 3D mesenchymal migration (20). In HIV-1-infected macrophages, the phosphorylation of WASP is enhanced and phosphorylated WASP accumulates at podosomes of infected cells in a Nef-dependent manner where it likely promotes the stability of F-actin (8). Thus, Nef controls the migration of macrophages by activating Hck, which in turn regulates the activity of WASP to stabilize podosomes and enhance mesenchymal migration (Figure 2).

The Nef/Hck axis mediates the increase in the mesenchymal migration of infected macrophages and probably their deleterious accumulation in tissues. In addition to the already proposed disruption of Nef/Hck interaction as a potential antiviral strategy (59–63), we propose to target this interaction to specifically restore the migration parameters of HIV-1-infected macrophages. Although it is well established that Nef is essential for AIDS pathogenesis, it is not currently targeted by antiviral therapeutic strategies. The first Nef inhibitor described in the literature that contains an optimized derivative of the SH3 domain of Hck, alters Nef interactions with SH3-bearing proteins (63). Moreover, small Nef-interacting proteins composed of a Nef-targeted SH3 domain fused to a sequence motif of the CD4 cytoplasmic tail and combined with a prenylation signal for membrane association have also been developed (61). Finally, Neffins have recently been developed. They comprise an anti-Nef single-domain antibody fused to part of the Hck SH3 domain (Neffins B6 and C1), inhibit all key activities of Nef in T cells, and inhibit cell fusion and modulation of podosomes in macrophages, two processes that depend on Hck activation by Nef (64, 65). These observations support the strategy to develop new antiviral drugs targeting Nef/Hck interaction.

## Hck: A Promising Pharmacological Target to Limit Macrophage Tissue Infiltration

Although macrophages play a key role in immune protection, they also play detrimental roles in several diseases including cancer and chronic inflammations. Elucidation of the migration modes and mechanisms used by macrophages was a challenge of the past decade, including the need to identify key migration players for pharmacological inhibition. One example is Hck, a migration effector that we postulate as a good target. Indeed, based on several studies involving its deletion/depletion in macrophages, Hck appears to be instrumental for podosome structure/function and mesenchymal migration *in vitro* and *in vivo* (8, 21–27, 45, 57). Of note, Hck expression is restricted to phagocytes and thus its pharmacological inhibition should not impact other cell types. Furthermore, HIV-1 developed the strategy to activate Hck to enhance the mesenchymal migration of macrophages (8), further supporting Hck as a therapeutic target. Actually, the virus teaches us how to control a migration mode that is involved in macrophage infiltration in tumors (8, 21). Collectively, we believe that targeting Hck might have strong implications in controlling the macrophage recruitment in cancer and possibly in other diseases including AIDS.

## Conclusion

HIV-1 is able to modify the migration of its main host cells (T lymphocytes and macrophages) *via* Nef, which appears as a key regulator of the migration of T cells and the different modes of macrophage migration. This is a powerful pathogenic strategy that could favor cell-to-cell spreading of the virus by inhibiting T cell migration in lymph nodes and promoting virus dissemination through body tissues by enhancing macrophage 3D mesenchymal migration. Similarly to the actin-based motility of bacteria employed by *Listeria* or *Shigella*, a biological context that yielded major advances in the in-depth knowledge of the actin polymerization machinery (66), HIV-1 has similarly developed a strategy to exploit the actin cytoskeleton and cell migration, and this way, reveals substantial insights into molecular pathways regulating cell migration.

## Conflict of Interest Statement

The authors declare that the research was conducted in the absence of any commercial or financial relationships that could be construed as a potential conflict of interest.
